# Cucumber RDR1s and cucumber mosaic virus suppressor protein 2b association directs host defence in cucumber plants

**DOI:** 10.1111/mpp.13112

**Published:** 2021-08-06

**Authors:** Reenu Kumari, Surender Kumar, Diana Leibman, Bekele Abebie, Yulia Shnaider, Shou‐Wei Ding, Amit Gal‐On

**Affiliations:** ^1^ Department of Plant Pathology and Weed Research Agricultural Research Organization Rishon LeZion Israel; ^2^ College of Horticulture and Forestry Dr YS Parmar University of Horticulture and Forestry Mandi India; ^3^ Plant Virology Lab, Biotechnology Division CSIR‐Institute of Himalayan Bioresource Technology Palampur India; ^4^ Department of Plant Pathology and Microbiology & Institute for Integrative Genome Biology University of California Riverside California USA

**Keywords:** cucumber mosaic virus suppressor 2b, cucumber RDR1, host defence, protein‒protein interaction, protoplast

## Abstract

RNA‐dependent RNA polymerases (RDRs) regulate important aspects of plant development and resistance to pathogens. The role of RDRs in virus resistance has been demonstrated using siRNA signal amplification and through the methylation of viral genomes. Cucumber (*Cucumis sativus*) has four *RDR1* genes that are differentially induced during virus infection: *CsRDR1a*, *CsRDR1b*, and duplicated *CsRDR1c1*/*c2*. The mode of action of CsRDR1s during viral infection is unknown. Transient expression of the cucumber mosaic virus (CMV)‐2b protein (the viral suppressor of RNA silencing) in cucumber protoplasts induced the expression of *CsRDR1c*, but not of *CsRDR1a/1b*. Results from the yeast two‐hybrid system showed that CsRDR1 proteins interacted with CMV‐2b and this was confirmed by bimolecular fluorescence complementation assays. In protoplasts, CsRDR1s localized in the cytoplasm as punctate spots. Colocalization experiments revealed that CsRDR1s and CMV‐2b were uniformly dispersed throughout the cytoplasm, suggesting that CsRDR1s are redistributed as a result of interactions. Transient overexpression of individual *CsRDR1a/1b* genes in protoplasts reduced CMV accumulation, indicating their antiviral role. However, overexpression of CsRDR1c in protoplasts resulted in relatively higher accumulation of CMV and CMVΔ2b. In single cells, CsRDR1c enhances viral replication, leading to CMV accumulation and blocking secondary siRNA amplification of CsRDR1c by CMV‐2b protein. This suggests that CMV‐2b acts as both a transcription factor that induces CsRDR1c (controlling virus accumulation) and a suppressor of CsRDR1c activity.

## Graphical abstract

Cucumber mosaic virus 2b interferes with functioning of cucumber RDR1s (CsRDR1a/CsRDR1b/CsRDR1c) by direct interaction and induces transcription of *CsRDR1c* to exponential‐fold to control virus accumulation.

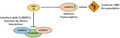

## 1 INTRODUCTION

Viruses pose serious threats to economically important crops by reducing yield and quality. Plants employ a variety of defence strategies to counter viral infection processes. RNA silencing, also known as RNA interference (RNAi), is a host defence mechanism that provides antiviral resistance/immunity by targeting the viral genome. RNAi degrades the viral genome into small RNAs (20–24 nucleotides [nt]), known as small interfering RNAs (siRNA), using DICER‐like (DCLs) endonucleases in a sequence‐complementarity manner (Baulcombe, 2004; Ding & Voinnet, 2007; Pumplin & Voinnet, 2013; Wang et al., 2010).

To achieve potent silencing, host RNA‐dependent RNA polymerases (RDRs) are crucial for manifestations of the RNAi process as they amplify viral double‐stranded (ds) RNA and transmit silencing signals (Ahlquist, 2002; Qu, 2010; Vance & Vaucheret, 2001). RDRs are characterized by the presence of a catalytic DLDGD domain and can synthesize dsRNA from single‐stranded (ss) RNA templates by primer‐independent and primer‐dependent mechanisms (Willmann et al., 2011). The resulting dsRNA is then reincorporated into a cyclical RNA degradation pathway, in which RNA silencing leads to the amplification of silencing signals for the degradation of the target RNA (Lipardi et al., 2001; Sijen et al., 2001; Wang et al., 2010). These RDRs are classified into three major clades: RDRα, RDRβ, and RDRγ; plants contain only RDRα and RDRγ (Zong et al., 2009). The *Arabidopsis* genome encodes six different RDRs, of which RDR1, RDR2, and RDR6 play important roles in the biogenesis of small RNA (Willmann et al., 2011). RDR1 and RDR6 are known to play important roles in antiviral immunity by producing viral dsRNA (Guo et al., 2019) and preventing meristem invasion (Schwach et al., 2005). Recent studies have identified two host factors, antiviral RNAi‐defective 1 (AVI1) and AVI2, that enhance the activity of RDR1 and RDR6 for the generation of viral siRNAs (Guo et al., 2017, 2018). Moreover, RDR1/RDR6 loss‐of‐function mutants show increased susceptibility to many RNA viruses (Guo et al., 2018; Qi et al., 2009; Wang et al., 2010). After infection by cucumber mosaic virus (CMV) or turnip mosaic virus, RDR1 was found to be responsible for the biogenesis of a distinct class of endogenous virus‐activated siRNAs that confer broad‐spectrum antiviral responses by silencing host genes (Cao et al., 2014). RDR2 plays an important role in the biogenesis of host‐derived 24‐nt repeat‐associated siRNAs (rasiRNAs) (Garcia‐Ruiz et al., 2010).

CMV is a widespread pathogenic member of the *Bromoviridae* family with a broad host range that includes many economically important crops (Palukaitis & García‐Arenal, 2003). Its genome consists of three positive‐sense, ssRNA segments (RNAs 1 to 3), that together encode five proteins. The 1a and 2a proteins, which are encoded by RNA‐1 and RNA‐2, form part of the replication complex that is responsible for the replication of the viral genome. The 2b protein is encoded by a subgenomic RNA (RNA‐4A) derived from RNA‐2 (Ding et al., 1994) and is a multifunctional protein that acts as a viral suppressor of RNA silencing (VSR; Diaz‐Pendon et al., 2007) and aids the long‐distance movement of the virus and symptom induction (Ding et al., 1995). RNA‐3 encodes two proteins, movement protein (MP) that translates from the 5′ open reading frame and is responsible for cell‐to‐cell virus movement, and the 3′ open reading frame that translates the viral capsid or coat protein (CP) through subgenomic RNA‐4 (Palukaitis & García‐Arenal, 2003).

To evade the host defence machinery, viruses encode VSR proteins, which can interfere with the RNA silencing pathway at several points, to suppress this process (Csorba et al., 2015). The CMV‐2b protein (referred to hereafter as “2b”) is a well‐known VSR that obstructs posttranscriptional gene silencing and transcriptional gene silencing (Guo & Ding, 2002; Hamera et al., 2012). The 2b protein plays a role in binding both 21–24 nt siRNA duplexes and long (55 nt) dsRNAs (Duan et al., 2012). Argonaute (AGO) proteins are important for the gene‐silencing process. The 2b protein has been shown to interact with the AGO PAZ and PIWI domains, interfering with AGO functions (Duan et al., 2012; Hamera et al., 2012; Zhang et al., 2006). An earlier report by Hamera et al. (2012) showed that, during virus infection, 2b interferes with genome methylation or transcriptional gene silencing and changes the expression profile of many *Arabidopsis* genes. In addition, Duan et al. (2012) demonstrated the remodelling of the *Arabidopsis thaliana* genome methylation pattern due to the binding of the 2b protein with 21‐, 22‐, or 24 nt siRNA. Transgenic *A*. *thaliana* plants expressing the 2b protein show a genome‐wide reduction of CHH and CHG methylation in their transposable elements and coding genes (Hamera et al., 2012; Zhao et al., 2016). However, this suppression of DNA methylation is independent of the AGO‐binding activity of the 2b protein (Duan et al., 2012). The 2b protein also plays an important role in symptom development and the systemic movement of CMV (Lewsey et al., 2010; Wang et al., 2004).

Interaction of the 2b protein with *Arabidopsis* catalase 3 decreases catalase activity (specifically the scavenging of cellular hydrogen peroxide), causing necrotic symptom development during CMV infection and thus favouring virus infection (Inaba et al., 2011). A CMVΔ2b mutant lacking the *2b* gene was reported to accumulate at high levels along with severe symptoms in *Arabidopsis RDR1* or *RDR6* loss‐of‐function mutants (*rdr1*, *rdr1/rdr6,* and *rdr1/rdr2/rdr6*), indicating the importance of the *RDR1/RDR6* genes for immunity to CMV (Wang et al., 2010). Recently, it was found that autophagy reduces CMV accumulation via 2b degradation and contributes to resistance that is mediated by AGO1‐dependent and DCL1/4‐dependent RNA‐silencing mechanisms (Shukla et al., 2020).

Most plant families encode single or duplicated *RDR1* genes (Chen et al., 2010; Qin et al., 2017; Willmann et al., 2011); exceptionally, the cultivated Cucurbitaceae encode a number of putative *RDR1* genes (Leibman et al., 2018). The cucumber (*Cucumis sativus*) genome encodes four *RDR1* genes, namely *CsRDR1a*, *CsRDR1b*, and duplicated *CsRDR1c1*/*c2*, that have been shown to be differentially induced during infection by various viruses (Leibman et al., 2018).

These studies demonstrate the role of the 2b protein in pathogenicity, based on its suppression of a variety of RNA‐silencing activities. In this study, the role of RDR1s in defence activities against CMV infection was evaluated in cucumber and we explored the exact roles of individual *RDR1* genes during virus infection. In the present study, the hypothesis of association between cucumber CsRDR1s and the 2b protein, and the impact of that interaction on the progress of CMV infection, were evaluated using various assays. This study demonstrates the interaction and localization of the 2b with CsRDR1s, despite suggesting differential regulation of CsRDR1s. Higher levels of expression of *CsRDR1*s can effectively suppress virus infection and associated symptoms to aid the development of tolerance/resistance.

## 2 RESULTS

### 2.1 CMVΔ2b failed to induce *CsRDR1* expression

**FIGURE 1:**
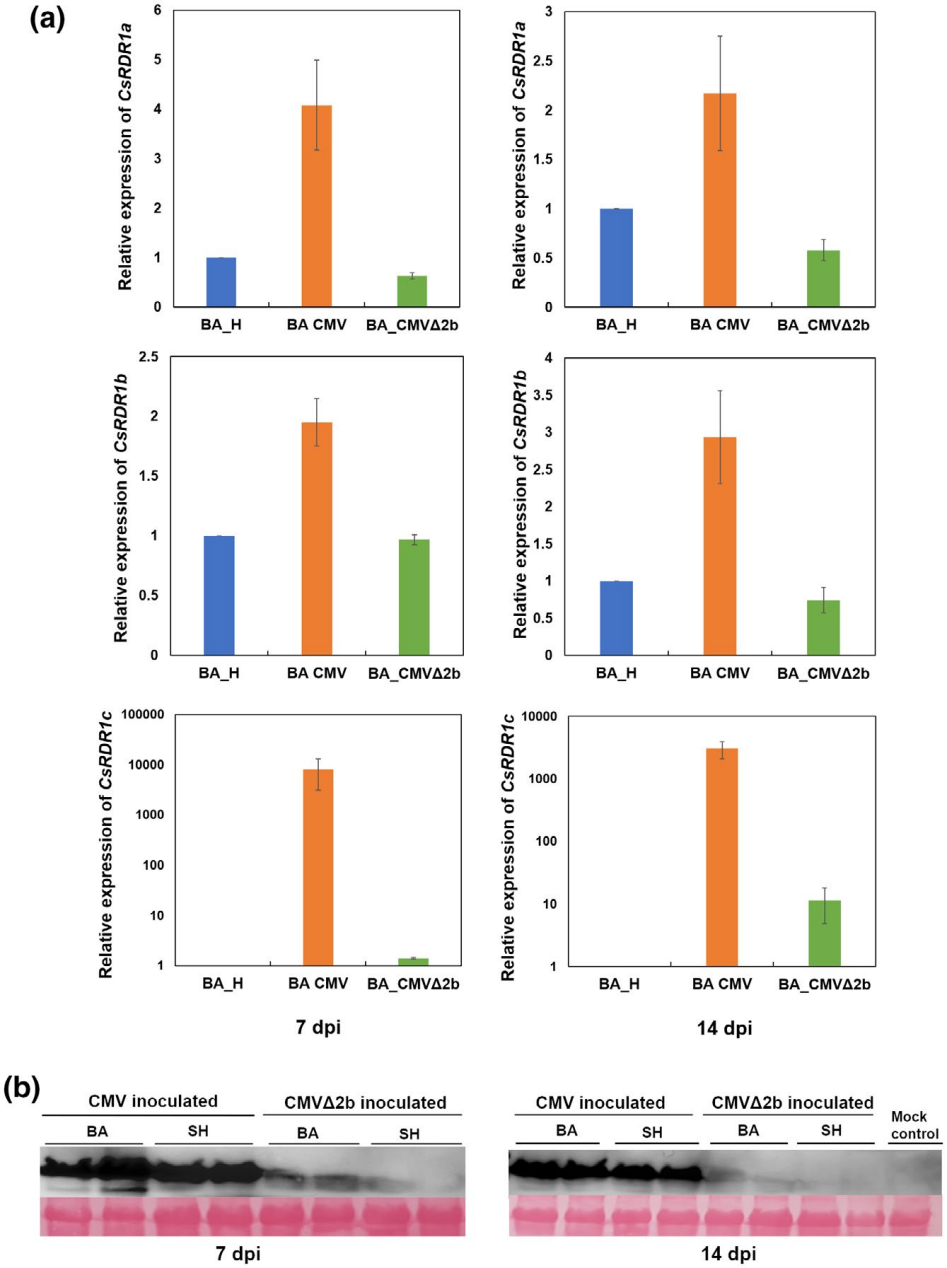
(a) Expression levels of *CsRDR1*s (*CsRDR1a*, *CsRDR1b*, and *CsRDR1c*) in cv. Bet Alfa (BA) at 7 and 14 days postinoculation (dpi) with CMV or the CMVΔ2b mutant. The mRNA levels of *CsRDR1a*, *CsRDR1b*, and *CsRDR1c* were calculated with reference to mock inoculated controls (H) for the respective time intervals. The expression levels were normalized to *EF‐1α* and relative expression was calculated using the 2^−ΔΔ^ *^C^* ^t^ method. Three biological replicates were taken for analysis and each sample was a pool of three plants. Error bars represent the mean ± *SD* (*n* = 3). Blue bars represent mock control samples, orange bars represent CMV‐infected samples, and green bars represent CMVΔ2b‐infected samples. (b) Immunodetection of CMV coat protein (CP) in CMV‐ and CMVΔ2b‐inoculated cv. Bet Alfa and cv. Shimshon (SH) plants at 7 and 14 days postinoculation (dpi) using CP‐specific antiserum (1:3,500 dilution). Ponceau staining is shown for equal sample loading

Cucurbits possess a unique *RDR1* family comprising four genes (*RDR1a*, *RDR1b*, *RDR1c1*, and *RDR1c2*), which are differentially expressed during virus infection (Leibman et al., 2018). Here, we investigated the role of cucumber *RDR1*s (*CsRDR1*s) during infection by CMV and a 2b‐deficient CMV mutant (CMVΔ2b) in cucumber cv. Bet Alfa, a very virus‐susceptible cultivar (Wang et al., 2003). To produce the CMVΔ2b clone, the RNA‐2 biologically active cDNA clone was modified. Specifically, two nucleotide changes were made in each of the first three ATG codons (ATG→ACC) of the *2b* gene, as a CMVΔ2b clone with only one nucleotide change (ATG→ACG) reverted to wild type in cucumber. Thus, *2b* gene expression was disrupted without affecting 2a protein translation. During CMV infection, expression of *CsRDR1a* remained higher (c.4‐fold at 7 days postinoculation [dpi]) in infected cv. Bet Alfa plants than in healthy plants; that difference decreased to c.2‐fold at 14 dpi (Figure 1a). *CsRDR1b* expression levels were increased up to 2‐fold at 7 dpi and 3‐fold at 14 dpi during CMV infection, relative to healthy control plants (Figure 1a). In contrast, *CsRDR1a* and *CsRDR1b* expression levels remained unchanged (or slightly decreased) in CMVΔ2b‐infected cv. Bet Alfa plants (Figure 1a). Duplicate versions of *CsRDR1c* (*CsRDR1c1* and *CsRDR1c2*) with 98% identity were observed in infected plants but were not expressed in healthy plants. Previously, *CsRDR1c1* and *CsRDR1* *c2* were found to be similarly expressed during virus infection (Leibman et al., 2018), so expression of *CsRDR1c1* alone was examined and hereafter *CsRDR1c1+2* is referred to as *CsRDR1c*. In contrast to the results obtained for *CsRDR1a/1b*, very high levels of *CsRDR1c* expression were observed on CMV infection, with an 8,000‐fold increase at 7 dpi and a 3,000‐fold increase at 14 dpi in cv. Bet Alfa plants (Figure 1a).

During CMVΔ2b infection, *CsRDR1a* and *CsRDR1b* expressions were not induced. The level of *CsRDR1c* expression also remained unchanged at 7 dpi, but it showed a 10‐fold increase at 14 dpi relative to healthy cv. Bet Alfa plants. These results demonstrate that *CsRDR1a*, *CsRDR1b*, and *CsRDR1c* expression change is associated with either the CMV level or presence of the 2b protein, as no or less induction was observed during CMVΔ2b infection as compared to CMV (at 7 and 14 dpi; Figure 1a). Similar results were obtained in western blotting, where higher CMV coat protein (CP) accumulation was observed at 7 and 14 dpi in CMV‐infected cv. Bet Alfa as compared to CMVΔ2b mutant‐infected samples (Figure 1b). A similar trend was observed in a tolerant cucumber cv. Shimshon during CMV and CMVΔ2b infection (Figure 1b). These results clearly suggest that the expression of the CMV‐*2b* gene is important for the induced expression of *CsRDR1*a, *CsRDR1b*, and *CsRDR1c* during infection.

### 2.2 CMV‐2b enhances *CsRDR1c* expression in single cells

**FIGURE 2:**
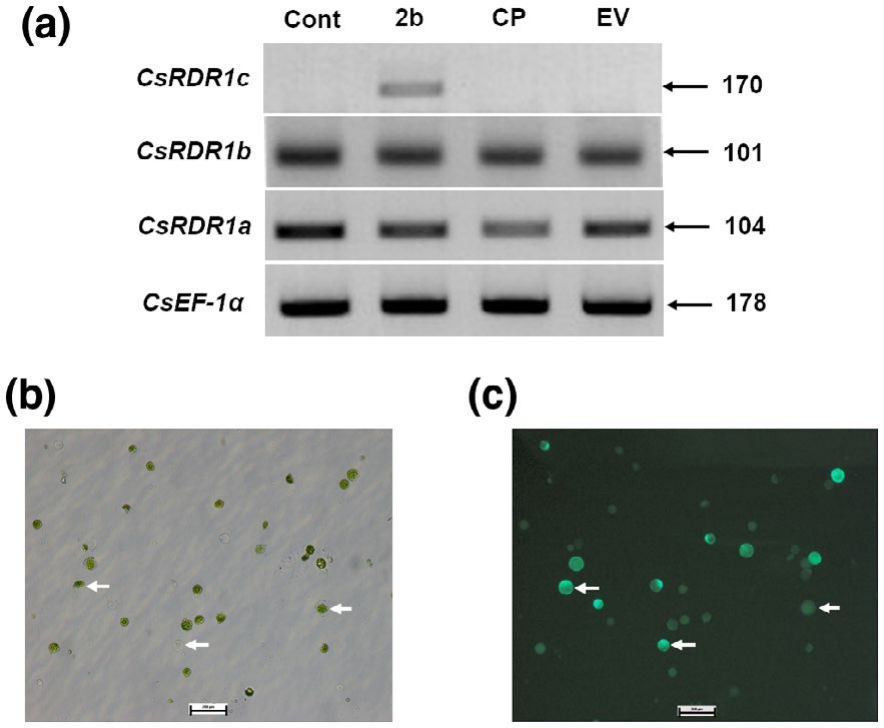
(a) *CsRDR1c* expression in cucumber protoplasts induced in the presence of CMV 2b. Cucumber protoplasts were transfected with pSAT6‐2b^GFP^ (2b) and pSAT6‐CP^GFP^ (CP) under the 35S promoter and levels of expression of *CsRDR1a*, *CsRDR1b*, and *CsRDR1c* were analysed at 72 hr posttransfection by reverse transcription PCR. The sizes of amplified PCR products are written next to arrows. Cucumber protoplasts treated with empty pSAT6^GFP^ vector (EV) or polyethylene glycol (PEG; Cont) served as controls. EF‐1α expression (*CsEF1α*) served as a housekeeping‐gene reference. Expression of the 2b protein fused to green fluorescent protein (GFP) in protoplasts was observed under (b) a bright field and (c) UV light (three protoplasts are marked with white arrows in both fields)

The role of the 2b protein in the induction of *CsRDR1s* was assessed by transient expression of the 2b protein in cucumber protoplasts. Cucumber protoplasts isolated from cv. Bet Alfa plants were transfected with the expression vectors pSAT6‐CMV^CP‐GFP^ (CP), pSAT6‐CMV^2b‐GFP^ (2b), and the pSAT6^EV‐GFP^ empty vector control (EV; Figure 2).

After 72 hr of incubation, the expression levels of endogenous *CsRDR1a*, *CsRDR1b*, and *CsRDR1c* were determined using reverse transcription (RT) PCR. It was observed that 2b protein itself could induce *CsRDR1c*, whereas CP overexpression had no such effect (Figure 2). However, the *CsRDR1b* and *CsRDR1a* genes were expressed in cucumber leaf protoplasts and the levels of their expression were not affected by the expression of 2b or CP, as compared to polyethylene glycol (PEG)‐treated controls (Cont) and pSAT‐EV (empty vector, EV) (Figure 2). These results indicate that the 2b protein that was localized to the nucleus acted directly or indirectly as an inducer (“transcription factor”) for *CsRDR1c* regulation.

### 2.3 Effect of *CsRDR1* overexpression against CMV at the single‐cell level

**FIGURE 3:**
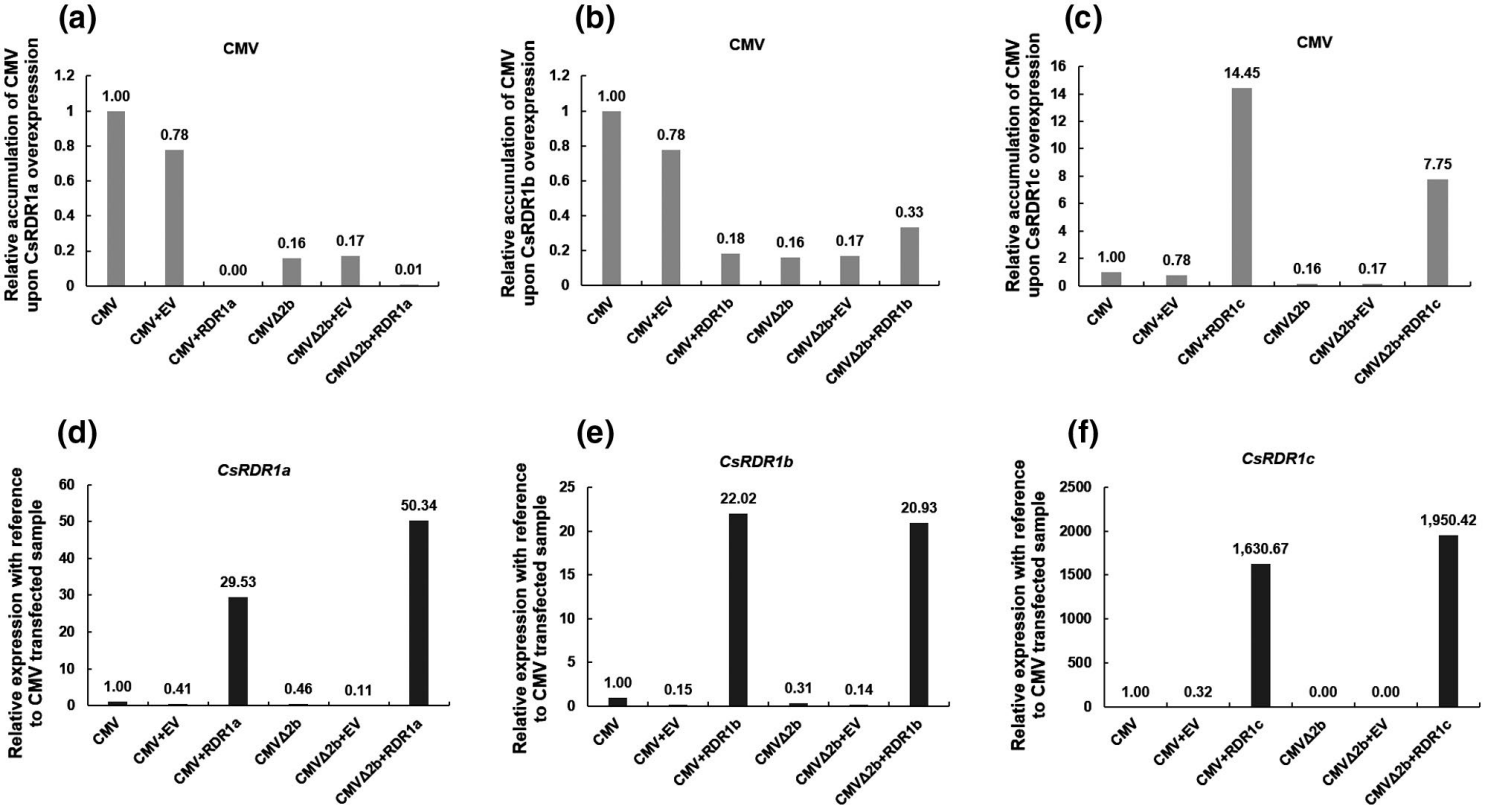
Effects of CsRDR1s on the accumulation of CMV and CMVΔ2b in cucumber protoplasts. (a–c) CMV and CMVΔ2b accumulation in cucumber protoplasts transfected with pSAT6‐eCFP constructs of CsRDR1a^eCFP^, CsRDR1b^eCFP^, and CsRDR1c^eCFP^ or empty pSAT6‐eCFP vector (EV) along with in vitro transcripts of CMV or CMVΔ2b mutant. CMV and CMVΔ2b levels were determined by reverse transcription (RT) PCR of cucumber protoplasts at 68 hr after transfection. Virus accumulation was calculated with reference to CMV‐transfected control samples. (d–f) Accumulation of *CsRDR1a*, *CsRDR1b*, and *CsRDR1c* was verified by quantitative RT‐PCR and normalized to that of *EF‐1α*. The experiment was repeated twice with similar results and data from one of the replicates is shown

To investigate the activity of CsRDR1s against CMV, *CsRDR1a*/*1b*/*1c* were expressed transiently in cucumber protoplasts, along with CMV in vitro transcripts. Cucumber protoplasts transfected with CMV, CMV+pSAT6‐EV^GFP^, and nontransfected protoplasts were used as controls. Relative CMV accumulation was determined 68 hr posttransfection by quantitative RT‐PCR (RT‐qPCR; Figure 3). Transient expression of *CsRDR1a* effectively suppressed the accumulation of CMV and CMVΔ2b compared to control protoplasts transfected with either CMV alone or CMV+pSAT6‐EV^GFP^ (Figure 3a). Similarly, transiently expressed *CsRDR1b* effectively suppressed the accumulation of CMV about 6‐fold. However, a smaller increase in CMVΔ2b accumulation (c.2‐fold) was observed (Figure 3b). In contrast to *CsRDR1a* and *CsRDR1b*, transient expression of *CsRDR1c* was associated with increased accumulation of CMV (close to 14‐fold), whereas a c.7‐fold increase was observed for the CMVΔ2b mutant (Figure 3c). The ectopic expression of the *CsRDR1*s was confirmed by RT‐qPCR with reference to the CMV‐ and CMVΔ2b‐transfected protoplasts (Figure 3d‒f). Endogenous *CsRDR1*s expression remained lower during this period in cucumber protoplasts, which suggests that ectopically expressed *CsRDR1a* and *CsRDR1b* triggered an antiviral response against CMV by 68 hr posttransfection. Interestingly, transient expression of *CsRDR1c* during CMV infection was 54 and 74 times greater than the expression of *CsRDR1a* and *CsRDR1b*, respectively, and 39 and 97 times greater during CMVΔ2b infection than for *CsRDR1a* and *CsRDR1b*, respectively (Figure 3d‒f). This highly increased level of *CsRDR1c* expression in protoplasts is probably due to autopositive regulation of the endogenous *CsRDR1c* expression in the presence of CMV, as was observed in cucumber leaves (Figure 1). The unexpected increase in the levels of CMV and CMVΔ2b due to transient expression of *CsRDR1c* (Figure 3c), in contrast to transient expression of *CsRDR1a* or *CsRDR1b*, suggests that *CsRDR1c* abundance alone in CMV‐infected cells plays a role in enhancing viral replication at the cell level for a short time posttransfection.

### 2.4 2b protein, a viral suppressor of RNA silencing, directly interacts with CsRDR1 proteins in yeast

**FIGURE 4:**
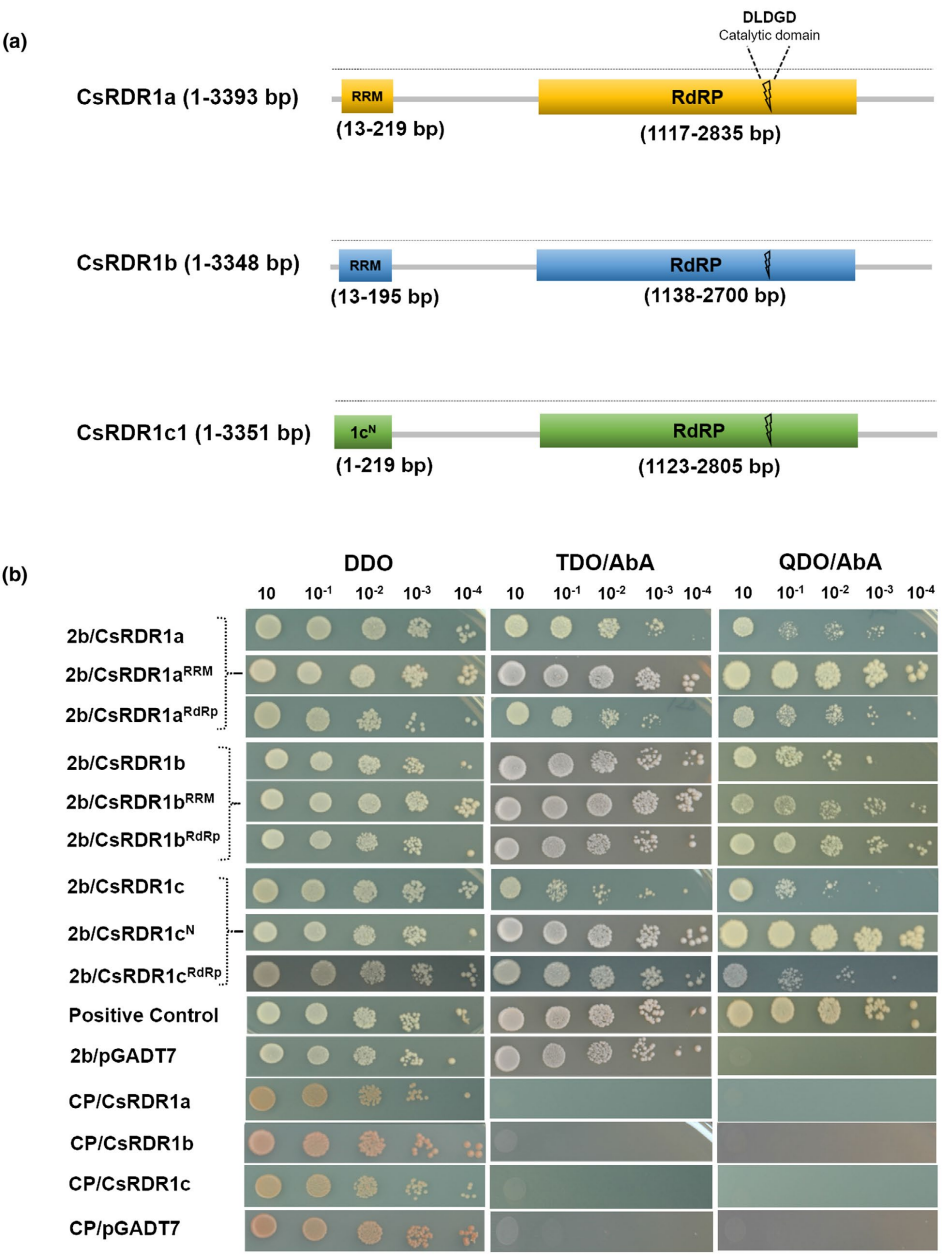
CMV‐2b displayed association with CsRDR1a/1b/1c. (a) Schematic representation of RMM, N′(1c^N^), and RdRp regions of cucumber RNA‐dependent RNA polymerases 1: CsRDR1a, CsRDR1b, and CsRDR1c1, used to check the interaction with the 2b protein. (b) Interaction of CMV (Fny strain) 2b protein with CsRDR1s in yeast cells. The 2b protein was fused to the GAL4 DNA‐binding domain (DNA‐BD) and expressed via yeast strain Y2H Gold. Full‐length cucumber RDR1 proteins (CsRDR1a, CsRDR1b, and CsRDR1c1) and domain regions of those proteins (RRM, N′ and RdRp) were expressed fused with the GAL4 activation domain (AD) in the Y187 yeast strain. Yeast mating was performed between 2b and CsRDR1 transformants and offspring were selected on double‐dropout selection (DDO) plates that were incubated at 28 °C for 3 days. The dilution assay was carried out on DDO, triple‐dropout (TDO), and quadruple‐dropout (QDO) selection plates at up to 10^−4^ dilution and cultures were kept for 3–4 days before imaging. The 2b protein, along with the empty vector control, and RDRs, along with CP, were used as negative controls

Because the *2b* gene was found to induce *CsRDR1c* expression (Figure 2), the possibility of a direct interaction of the 2b protein with CsRDR1 proteins was evaluated using the yeast two‐hybrid (Y2H) GAL4‐based Gold system. The 2b protein was used as bait to screen against three cucumber prey proteins: CsRDR1a, CsRDR1b, and CsRDR1c1. In general, RDRs are characterized by an RNA recognition motif (RRM) at the N‐terminus and the RNA‐dependent RNA polymerase catalytic domain (RdRp) in the centre of the protein (Figure 4). Therefore, RRM and RdRp regions were selected from CsRDR1a and CsRDR1b to generate prey constructs. The RRM region is not well defined in CsRDR1c1 and therefore a region (Nʹ) corresponding to that of other CsRDR1s was selected from the CsRDR1c1 N‐terminus (Figure 4a). The CMV 2b interacted strongly with CsRDR1a, CsRDR1b, and CsRDR1c (up to 10^−3^/10^−4^ OD dilutions in quadruple‐dropout selection medium), whereas negative controls (empty bait vector, pGBKT7, and pGBKT7‐Lam) showed no growth on triple/quadruple‐dropout selection plates with any CsRDR1 proteins (Figure 4b). The binding between 2b and the RRM of CsRDR1a/b and N′ of CsRDR1c was stronger than with the RdRp motif or the intact CsRDR1 proteins. Therefore, these three CsRDR1 proteins are able to interact with 2b in yeast cells. The specificity of these interactions was confirmed by testing other CMV proteins (i.e., CP [Figure 4b] and movement protein [MP; Figure S1]) as negative control bait proteins. None of the additional viral proteins had any interaction with CsRDR1s, as no growth was observed on selection medium plates (Figures 4b and S1). Thus, it was observed that CMV 2b protein specifically bound the CsRDR1a/1b/1c proteins in yeast cells.

**FIGURE 5:**
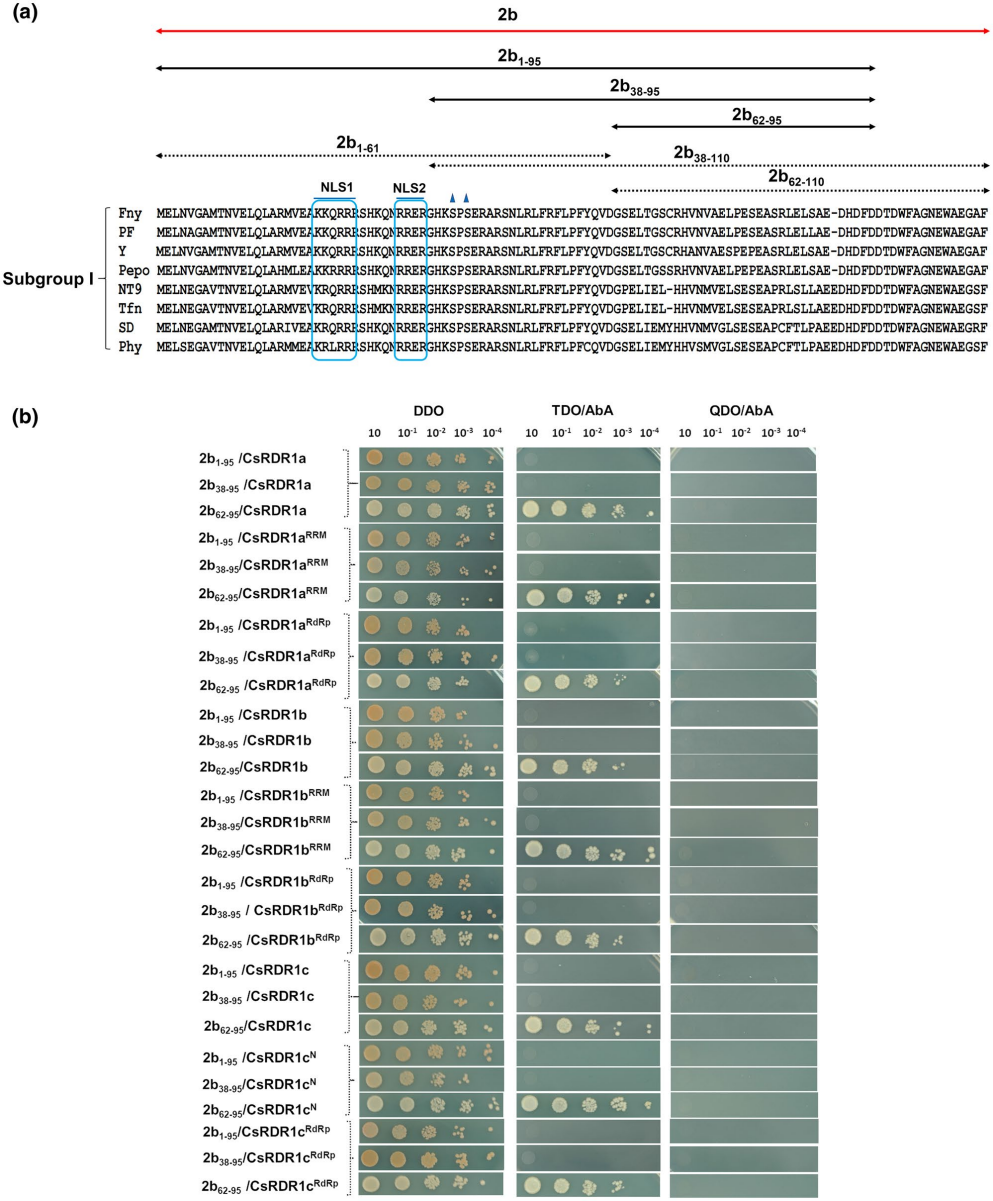
(a) Schematic representation of important domains in the 2b protein (CMV‐Fny strain; marked with black arrows) that were used for yeast two‐hybrid (Y2H) analysis. 2b protein sequences from Subgroup I were aligned to analyse conserved domains. 2b_1‐61,_ 2b_38‐110_, 2b_62‐110_ 2b_1‐95_, 2b_38‐95_, and 2b_62‐95_ represent the selected target regions for bait constructs. The 2b_1‐61_ domain includes nuclear localization signals NLS1 and NLS2 (marked with blue lines), and phosphorylation sites (shown by blue triangles). (b) Interaction of CMV‐Fny 2b protein deletion mutants with CsRDR1s, both full‐length and specific domains, in yeast cells. CMV 2b protein fragments were fused to the GAL4 DNA‐binding domain (DNA‐BD). Full‐length cucumber RDR1 proteins (RDR1a, RDR1b, and RDR1c) and specific domain regions (RNA recognition motif, RRM; RNA polymerase domain, RdRp) were expressed as fusions with the GAL4 activation domain (AD). After yeast mating, diploid transformants were selected on double‐dropout selection (DDO) plates at 28 °C for 3 days. Then a dilution assay was performed on double‐dropout (DDO), triple‐dropout (TDO), and quadruple‐dropout (QDO) selection plates (supplemented with the antibiotic aureobasidin A) involving up to 10^−4^ dilutions. Plates were kept for 3‒4 days before imaging. The 2b protein, along with empty vector control, and CsRDR1s were used as negative controls

We also identified different regions of the 2b protein that are important for interactions. The 2b protein has distinct domains for different functions: amino acids 1–61 have nuclear localization signals (NLS), a dsRNA/siRNA‐binding region and phosphorylation sites, the amino acid region 38–110 binds AGO proteins, and the region containing amino acids 62–110 lacks AGO‐binding affinity (Figure 5a). Therefore, various 2b protein deletion AD‐fusions (pGADT7), named 2b_1‐61_, 2b_1‐95_, 2b_38‐95_, 2b_38‐110_, 2b_62‐95_, and 2b_62‐110_, were generated to check binding with CsRDR1s (Figure 5b). In this study, it was found that CsRDR1s were able to interact with only a full‐length 2b protein (Figure 4b) and that deletion of the 2b C‐terminal region abolished the interaction with CsRDR1s, as no interaction was found with the 2b_1‐61_, 2b_1‐95_, 2b_38‐95_, or 2b_62‐95_ baits (Figure 5b). Thus, the 2b C‐terminus region was shown to be essential for the interaction in yeast (2b‐CsRDR1s). We were unable to use 2b_38‐110_ and 2b_62‐110_ as bait due to their autoactivation in yeast cells (Figure S2). In contrast, full‐length 2b, 2b_1‐61_, 2b_1‐95_, 2b_38‐95_, and 2b_62‐95_ did not show any autoactivation in the GAL4‐based Matchmaker Gold Y2H system (Figure S2). CsRDR1’s RRM and RdRp domains showed stronger affinity for the 2b protein than did full‐length CsRDR1s (Figure 4b). This may be due to failure of exposure of important motifs due to improper folding of full‐length proteins in yeast. However, no interaction was observed with the 2b C‐terminus deletion baits (2b_1‐61_, 2b_1‐95_, 2b_38‐95_, 2b_62‐95_; Figure 5b). These results demonstrate the importance of the 2b C‐terminus in this interaction, as summarized in Table 1.

**TABLE 1:** Summary of protein–protein interactions between 2b and CsRDR1s in yeast cells

	2bI^1–110^	2bI^1–95^	2bI^38–95^	2bI^62–95^	2bI^1–61^
CsRDR1a	+	−	−	−	−
CsRDR1a^RRM^	+	−	−	−	−
CsRDR1^RdRp^	+	−	−	−	−
CsRDR1b	+	−	−	−	−
CsRDR1b^RRM^	+	−	−	−	−
CsRDR1b^RdRp^	+	−	−	−	−
CsRDR1c	+	−	−	−	−
CsRDR1c^N^	+	−	−	−	−
CsRDR1c^RdRp^	+	−	−	−	−

### 2.5 CsRDR1s interact with CMV 2b in planta and are localized in the cytoplasm

**FIGURE 6:**
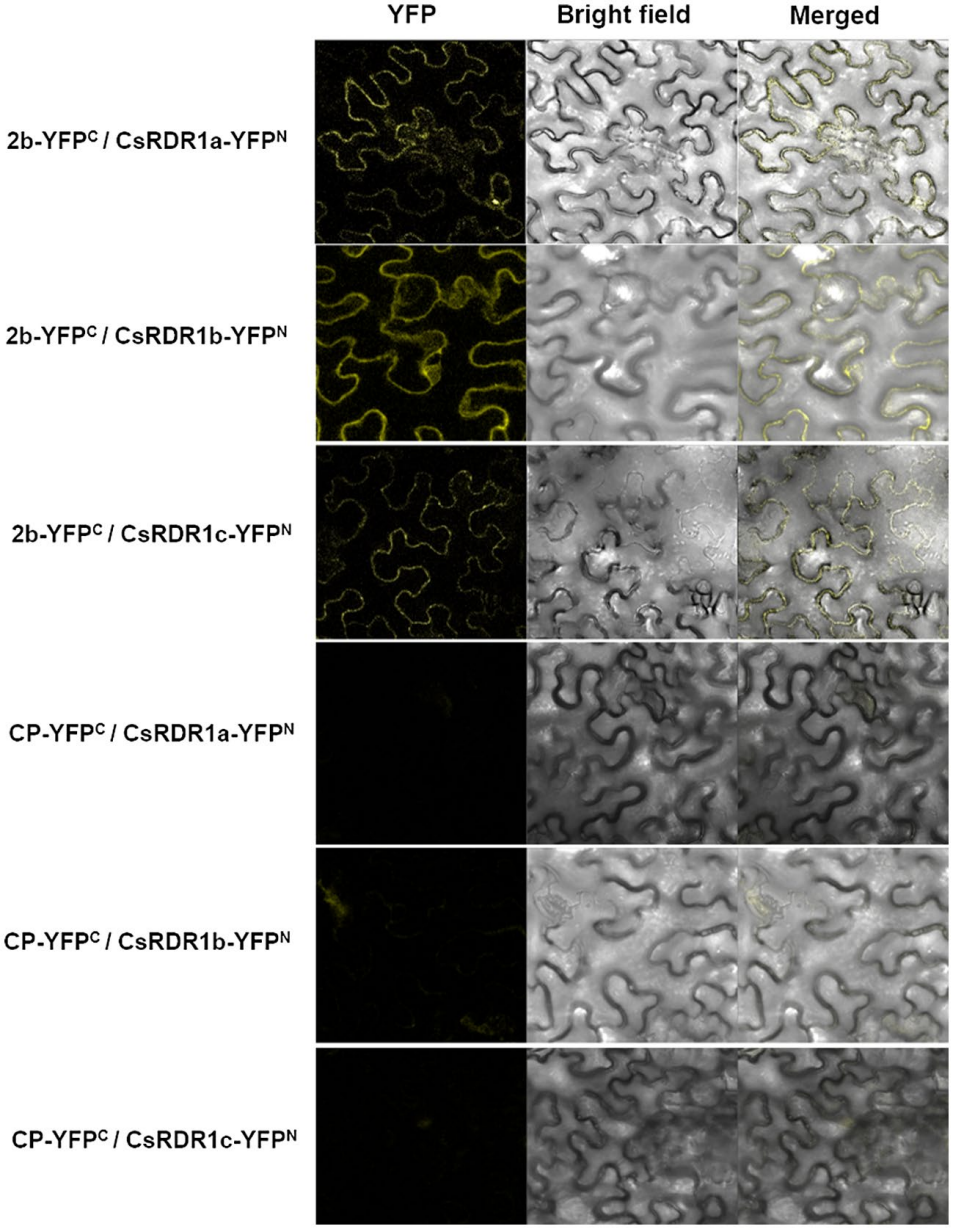
Bimolecular fluorescence complementation assay showing interaction between the CMV‐2b protein and cucumber RDR1 proteins (CsRDR1a, CsRDR1b, and CsRDR1c) in *Nicotiana benthamiana* leaf epidermal cells. 2b and CsRDR1 proteins were fused to the YFP^C^ terminus in the pSPYCE(M) vector and at the YFP^N^ terminus of the pSPYNE173R vector, respectively, and infiltrated in the following combinations: 2b‐YFP^C^/CsRDR1a‐YFP^N^, 2b‐YFP^C^/CsRDR1b‐YFP^N^, and 2b‐YFP^C^/CsRDR1c‐YFP^N^. CMV coat protein (CP) was also used as a negative control to analyse the interaction with CsRDR1 proteins in the following combinations: CP‐YFP^C^/CsRDR1a‐YFP^N^, CP‐YFP^C^/CsRDR1b‐YFP^N^, and CP‐YFP^C^/CsRDR1c‐YFP^N^

To verify the 2b–CsRDR1s interaction in planta, we performed bimolecular fluorescence complementation (BiFC) assays in *Nicotiana benthamiana* leaves. Growth media carrying *Agrobacterium tumefaciens* BiFC‐bearing constructs of the N‐ and C‐terminus halves of a yellow fluorescent protein (YFP), fused to the 2b or CsRDR1 coding sequences, were combined and agroinfiltrated into *N. benthamiana* leaves. Strong YFP reconstitution fluorescence was observed during CsRDR1a/1b/1c's interaction with 2b in the cytoplasm 48 hr postagroinfiltration (Figure 6). However, no YFP reconstitution was observed with an empty vector (not shown) or CMV CP, which were used as negative controls. These results, obtained using intact leaves, further confirm that 2b specifically associates with CsRDR1s.

### 2.6 CMV‐2b protein colocalizes with CsRDR1s in the cytoplasm of protoplasts

**FIGURE 7:**
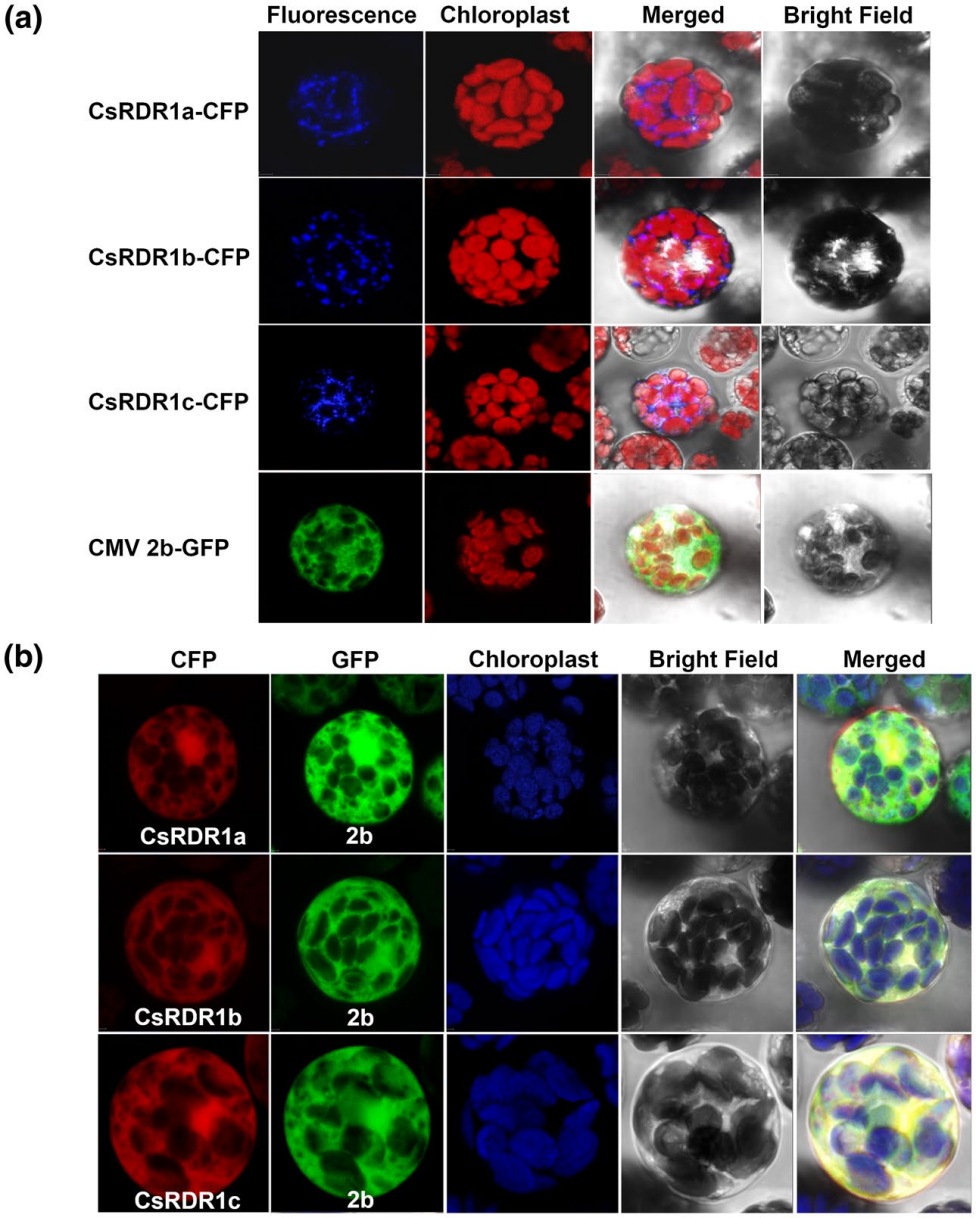
(a) Subcellular localization of CsRDR1a/1b/1c and CMV‐2b proteins in cucumber protoplasts, as viewed under a confocal laser scanning microscopy. CFP and GFP signals are shown in blue and green, respectively. Chloroplast autofluorescence is red. The CFP signals of CsRDR1a, CsRDR1b, and CsRDR1c proteins were observed in the cytoplasm as punctuate spots. The CMV 2b GFP signal was scattered throughout the cytoplasm and nucleus. (b) Colocalization of CsRDR1a‐eCFP, CsRDR1b‐eCFP, and CsRDR1c‐eCFP with CMV2b‐eGFP in cucumber protoplasts viewed under a confocal laser scanning microscope. Transient expression of CMV 2b and CsRDR1a, CsRDR1b, and CsRDR1c in cv. Bet Alfa cucumber protoplasts. CFP, GFP, and chloroplast autofluorescence are shown in blue, green, and red, respectively. Bright‐field images of the protoplasts and merged images of CFP and GFP fluorescence along with chloroplast autofluorescence are shown. The yellow colour indicates colocalization of CFP and GFP proteins in a merged image. Images were gathered 48 hr after transfection using a confocal scanning microscope

The subcellular localization of CMV 2b‐enhanced green fluorescent protein (2b‐eGFP) and CsRDR1s‐enhanced cyan fluorescent protein (CsRDR1a‐eCFP, CsRDR1b‐eCFP, and CsRDR1c‐eCFP) was examined in cucumber protoplasts after separate transfection. At 48 hr posttransfection, expression of 2b was observed in the nucleus and cytoplasm (Figure 7a), whereas CsRDR1a, CsRDR1b, and CsRDR1c were localized in the cytoplasm as granular structures (Figure 7a). The coexpression of these CsRDR1s with 2b was associated with colocalization of signals throughout the cytoplasm (Figure 7b), which was quite distinct from the localization of the individual CsRDR1s (Figure 7a), that is, coexpression led to cytoplasmic redistribution of these proteins.

## 3 DISCUSSION

Plant RDR1s are critical enzymes for RNA silencing that play a crucial role in the amplification of virus‐silencing signals. Natural and artificial RDR1 mutants display induced susceptibility to various viruses (Rakhshandehroo et al., 2009; Wang et al., 2010; Yang et al., 2004). To overcome RNA silencing‐mediated host resistance, viruses encode VSR proteins, which interfere with different steps of the RNA‐silencing mechanism (Burgyán & Havelda, 2011).

We have previously shown that the members of the cucumber *CsRDR1* gene family share 55%–60% homology. *CsRDR1a* and *CsRDR1b*, which are positioned in a head‐to‐head orientation, were expressed constitutively in healthy plants and a high level of *CsRDR1b* expression was associated with virus resistance in cucumber (Leibman et al., 2018). Here, we investigated the direct influence of the CMV silencing suppressor protein 2b on the expression of and interaction with members of the *CsRDR1* gene family in cucumber. First, we analysed the expression of the four *CsRDR1*s (*a*, *b*, *c1*, and *c2*) during infection by CMV and CMVΔ2b in cucumber. We observed that CMV infection increased *CsRDR1a* and *CsRDR1b* levels 2‐ to 4‐fold and increased *CsRDR1c* levels several thousand‐fold. We have shown previously that infection of cucumber with CMV and viruses belonging to different families also activates *CsRDR1c* transcription (Leibman et al., 2018). In the presence of the CMVΔ2b mutant, *CsRDR1a* and *CsRDR1b* levels did not change, but the *CsRDR1c* level increased c.10‐fold. In addition, in contrast to *CsRDR1a* and *CsRDR1b*, *CsRDR1c1/2* were not expressed in healthy cucumber or melon plants (data not shown). Therefore, based on the current study, we propose that higher levels of expression of *CsRDR1c* are dependent on either the virus titre or CMV 2b as reflected by CMV accumulation levels in plants (cv. Bet Alfa) that were infected with CMV or CMVΔ2b mutant. However, relative higher expression of *CsRDR1c* at 14 dpi despite lower virus titre compared to 7 dpi in CMVΔ2b mutant infected plants could be due to its accumulation with time after virus trigger or due to its autopositive regulation as observed in protoplasts on ectopic expression of *CsRDR1c*.

These results suggest that *CsRDR1c* has a different antiviral regulation and functional mechanism than either *CsRDR1a* or *CsRDR1b* and that the induced expression of *CsRDR1c* probably relies on the presence of the 2b protein. This mechanism must be absent from *CsRDR1a* and *CsRDR1b*. In *Arabidopsis*, AtRDR1 is regulated by a broad range of phytohormones (Hunter et al., 2013). Moreover, CMV infection induces and enriches plant immunity pathway genes, but not the expression of *AtRDR1* (Zhao et al., 2018), suggesting differences between the regulatory systems and that *CsRDR1c* plays a different role in cucumber than it does in *Arabidopsis*. Therefore, we examined the effect of 2b on the expression of genes from the *CsRDR1* family at the cellular level in cucumber protoplasts.

We showed, for the first time, that a suppressor like 2b can directly or indirectly induce *CsRDR1c* gene expression at the cellular level in protoplasts (after 68 hr) and in plants. Because 2b encodes two NLS motifs leading to nuclear localization (Wang et al., 2004) and has DNA‐binding abilities (Sueda et al., 2010), we propose that 2b acts like a transcription factor in the regulation of *CsRDR1c* gene expression.

CMV‐2b is a well‐known virulence factor that compromises host defences either by suppressing salicylic acid (SA)‐mediated resistance or by binding long/small dsRNAs, DNA, AGO proteins, and CMV 1a protein (Diaz‐Pendon et al., 2007; Ji & Ding, 2001; Lewsey et al., 2010; Watt et al., 2020; Zhao et al., 2018). This is why a CMVΔ2b mutant with a nonfunctional 2b protein is weaker in suppression of host defence and accumulates to only very low levels due to the slow systemic movement of the virus (Lewsey et al., 2009). Although 2b deletion was found to affect the initial rate of replication in protoplasts, the lack of 2b did not prevent the replication of a mutant virus, which later accumulated to similar levels (Soards et al., 2002).

In light of this, it is possible that 2b has dual activity: inducing *CsRDR1c* transcription to control virus accumulation and acting as a suppressor to block CsRDR1c activity. Previous studies have shown that the 2b protein inhibits the generation of secondary viral siRNA produced by RDR1 by binding CMV siRNAs and dsRNA precursors (Diaz‐Pendon et al., 2007), inhibiting the slicing activity of AGO1/4 by direct interaction with these proteins (Diaz‐Pendon et al., 2007; Fang et al., 2016; Hamera et al., 2012) and suppressing aphid resistance (Tungadi et al., 2020). Recently, CMV CP was also found to inhibit 2b and antagonize its viral suppressor function while maintaining a balance between virus accumulation and plant development (Zhang et al., 2017). Thus, the present study demonstrates a new role for 2b in virus self‐attenuation based on its control of *CsRDR1c* expression in cucumber.

The transient expression of CsRDR1a and CsRDR1b proteins in cucumber protoplasts resulted in the suppression of both CMV and CMVΔ2b titres, suggesting roles for those proteins in plant defence. This observation is also supported by earlier studies of the role of RDR1 in plant defence against CMV infection, in which RDR1 was shown to be important for the amplification of siRNAs from the 5′ region of CMV genomic RNA to enhance antiviral immunity (Wang et al., 2010). Later, RDR1 was also reported to play a role in biogenesis of virus‐activated siRNAs in *Arabidopsis*, which target endogenous genes during CMVΔ2b infection (Cao et al., 2014). The observation that CMV and CMVΔ2b RNA levels increase in cucumber protoplasts on *CsRDR1c* overexpression suggests that CsRDR1c encourages viral replication at the cell level and that the presence of the 2b protein is important for blocking CsRDR1c activity at the plant level. These data suggest that CsRDR1c might play a dual role: contributing to SA‐mediated antiviral defences and suppressing gene silencing. Similarly, overexpression of *Nt‐RDR1* in *N. benthamiana* enhanced virus hypersusceptibility, suggesting RDR6‐mediated antiviral RNA silencing (Ying et al., 2010). Earlier studies also showed that CMVΔ2b is unable to induce disease symptoms and demonstrated reduced virus accumulation (Lewsey et al., 2009). Thus, the present study presumes that, in addition to the CMV CP regulation of 2b (Zhang et al., 2017) and CMV 1a‐mediated regulation of the 2b–AGO1 association (Watt et al., 2020), the equilibrium between CMV‐2b and CsRDR1c protein levels controls virus accumulation and the infection progress.

Direct interaction was observed between CsRDR1s and the 2b protein. Previously, the 2b protein was shown to reduce the catalytic activity of AGO1 and AGO4 via direct interaction with their PIZI and PIWI domains to counter host defence activity (Hamera et al., 2012; Zhang et al., 2006). In the present study, the 2b protein directly associated with the RRM and RdRp domains (Shao & Lu, 2014; Willmann et al., 2011) of the CsRDR1a, CsRDR1b, and CsRDR1c proteins in yeast cells and in *N*. *benthamiana* leaves, as observed via BiFC assays. This interaction occurred only with the intact 2b protein, in contrast with the interaction with AGO proteins, and may indicate that the protein structure is essential for such contact. The 2b tetramer form is the biologically active moiety associated with siRNA duplexes (Gellért et al., 2012) and such a structure may be necessary for the interaction with RDR1 proteins.

In cucumber protoplasts, 2b was localized to the nucleus and also found throughout the cytoplasm; the CsRDR1a/1b/1c proteins were found throughout the cytoplasm in punctate spots. This finding is congruent with findings from previous studies of the 2b protein (Duan et al., 2012; Wang et al., 2004). Information about the subcellular distribution of RDR1 was not available previously and only RDR6 was known to be localized in cytoplasmic bodies (Kumakura et al., 2009). The colocalization of 2b and CsRDR1s in plant cells strengthens the assumption of a direct interaction between 2b and CsRDR1s, as demonstrated in the yeast system.

Coexpression of CMV‐2b and CsRDR1s in cucumber protoplasts resulted in the redistribution of these proteins throughout the cytoplasm into a very distinct pattern that differed from the pattern of the localization of the individual proteins. Moreover, redistribution of proteins in the presence of an interacting partner has also been observed in previous studies, specifically 2b coexpression with AGO1/AGO4 (Duan et al., 2012) and AtRDR6 with rice yellow stunt virus P6 protein (Guo et al., 2013). Nucleus/nucleolar localization of the 2b is essential for CMV virulence, while its cytoplasm‐localized form is more efficient in silencing suppression. The 2b protein partitions between the nucleus and cytoplasm, and thereby assists in maintaining the balance between CMV accumulation and injury to the host plant. Thus, 2b‐mediated redistribution of AGO1/4 to the nucleus benefits the virus by interfering with RDR‐dependent degradation of viral genomic RNA in the cytoplasm (Du et al., 2014). The present study suggests that CMV has evolved another approach to counter host antiviral defence: it directly interferes with host RDR1 activity to prevent secondary amplification of viral siRNAs. From a plant‐defence perspective, virus‐mediated induction of CsRDR1s in cucumber seems to play an important role in host defence against CMV by suppressing 2b virulence.

**FIGURE 8:**
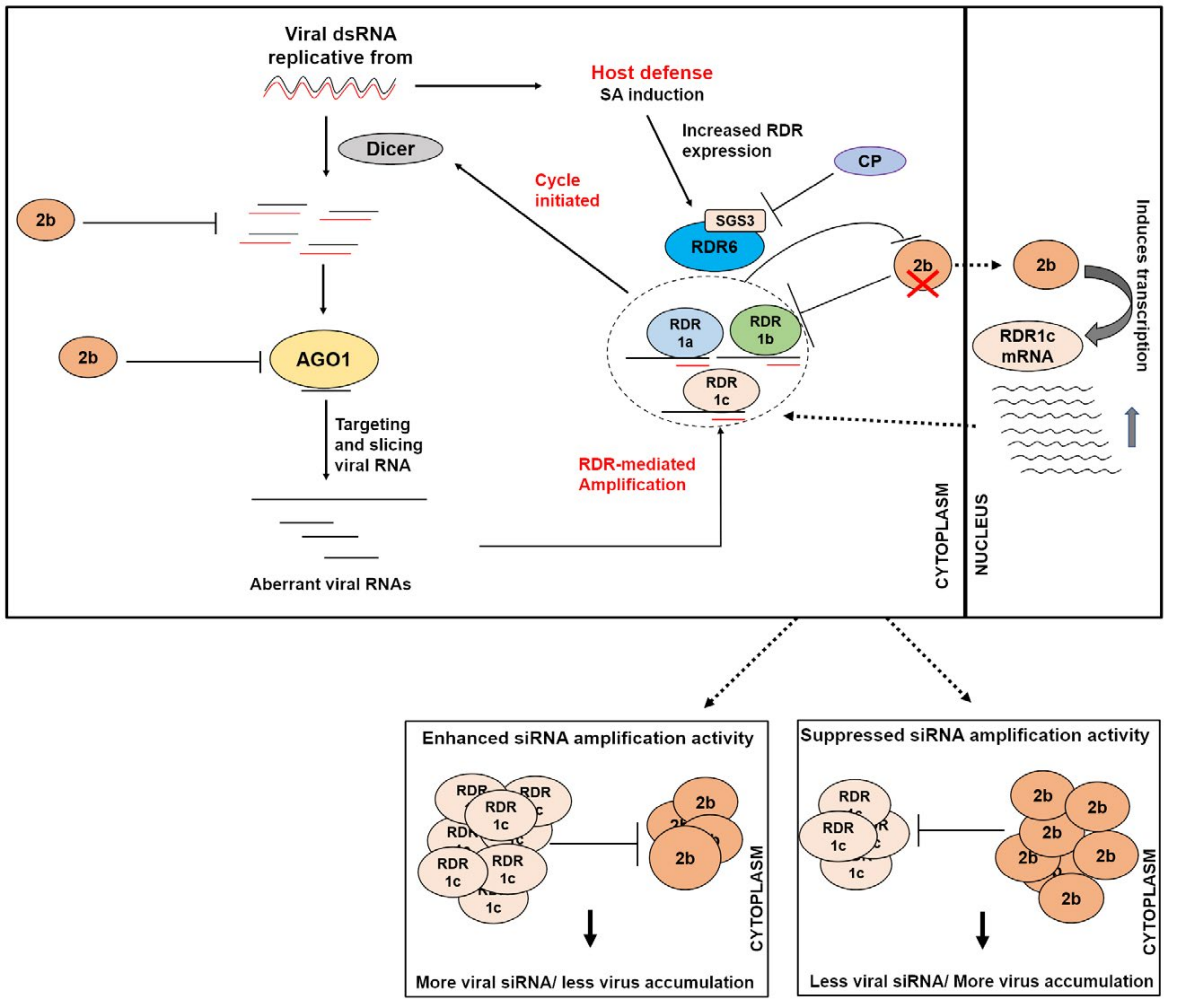
A model describing the regulation of the cucumber defence system as an equilibrium between CMV‐2b virulence and the RNA‐silencing activity of cucumber RDR1s. CMV‐2b interferes with cucumber RDR1s’ (1a, 1b, 1c) induction of antiviral silencing by directly binding these proteins and thereby interfering with the RDR1‐mediated viral siRNA amplification process. The presence of CMV‐2b also acts as a trigger for the induction of *CsRDR1c* expression. Hence, oscillations in the equilibrium between cucumber RDR1c and CMV‐2b protein levels direct virus accumulation. Only steps important for CMV pathogenicity are shown in this figure. dsRNA, double‐stranded RNA; AGO1, argonaute protein 1; RDR, RNA‐dependent RNA polymerase protein; SA, salicylic acid; 2b, CMV‐2b protein; CP, CMV coat protein

Taken together, these results present a dual role for the CMV‐2b protein (Figure 8). In one role, it physically interacts with host‐protein RNA‐silencing components (i.e., CsRDR1s) to interfere with the amplification of viral siRNAs. In the other role, 2b acts like a transcription factor with its DNA‐binding properties to affect the expression of *CsRDR1c*, and to balance plant growth and virus accumulation. These results also lead to the concept that higher induction of *CsRDR1a/1b/1c* genes during infection counters viral infection, but that CMV has evolved a strategy to counter this function that involves a 2b‐mediated interaction. Furthermore, it may be suggested that a shift in equilibrium between 2b and CsRDR1c protein levels directly affects virus accumulation and the infection progress.

## 4 EXPERIMENTAL PROCEDURES

### 4.1 Plant growth conditions and virus inoculations

*N. benthamiana* plants were grown in a growth chamber at 25 ℃ with a 14‐hr photoperiod. These plants were used for agroinfiltration at the three‐ to four‐leaf stage (Shiboleth et al., 2007). Cucumber cultivars Bet Alfa and Shimshon were maintained under growth conditions similar to those described above and plants at the cotyledon stage with an emerging first true leaf were inoculated mechanically with CMV‐Fny (Wang et al., 2003). For inoculum, CMV‐infected leaves were homogenized in 50 mM sodium phosphate buffer, pH 7.0, and mock controls were inoculated with buffer only. The samples were collected and frozen in liquid nitrogen until further analysis of virus titre. For protoplast isolation, the fully expanded first cucumber leaves (c.15 days after germination) from plants grown as above were used.

### 4.2 Construct preparation

To prepare Y2H bait constructs, PCR products of CMV‐Fny *2b* (full‐length and various domains), *CP*, and *MP* (accession D10538) were cloned into the pGBKT7 vector (Clontech) at the appropriate restriction sites (Table S1). Similarly, full‐length and domain regions of *CsRDR1a* (accession KT316427), *CsRDR1b* (accession KT316425), and *CsRDR1c1* (accession KT316426) were cloned into the pGADT7 vector (Clontech).

For the BiFC assay (Waadt et al., 2008), *2b* and *CP* were cloned at *Bam*HI/*Xho*I and *Bam*HI/*Sma*I sites, respectively, into the pSPYCE(M) vector to generate 2b‐pSPYCE and CP‐pSPYCE. *CsRDR1a/1b/1c1* were cloned at *Bam*HI/*Sma*I, *Bam*HI/*Xho*I, or *Bam*HI/*Sac*I sites in pSPYNE(R) 173 to produce CsRDR1a‐pSPYNE, CsRDR1b‐pSPYNE, and CsRDR1c‐pSPYNE, respectively. For subcellular localization in protoplasts, the *2b* and *CP* genes were cloned at *Nco*I/*Bam*HI sites in the pSAT6‐EGFP‐N1 vector (Chung et al., 2005). *CsRDR1a/1b/1c1* genes were cloned in the *Sal*I/*Bam*HI, *Xho*I/*Bam*HI, or *Eco*RI/*Bam*HI sites, respectively, in pSAT6‐ECFP‐C1.

### 4.3 Generation of CMV‐2b mutants

Infectious clones of CMV‐Fny (pFny109, pFny209, and pFny309; Rizzo & Palukaitis, 1990) and pFny209Δ2b were used to produce in vitro transcripts. pFny209Δ2b contains one nucleotide change in the first ATG of the coding frame, that is, ATG was changed to ACG (methionine to threonine), so that methionine (met1) would not be available for starting translation of the *2b* gene. However, we found that this CMVΔ2b reverts to wild type 7 dpi in cucumber, whereas the mutation was found to be stable in *N. benthamiana* and *N. tabacum*. Therefore, a new CMVΔ2b infectious clone (with the first three codons for methionine mutated ATG→ACC) was generated as described by Du et al. (2007); that mutation was stable in cucumber.

Briefly, overlap extension PCR was used to generate fragments that had terminal *Hin*dIII and *Pst*I restriction sites for subsequent cloning into the pFny209 plasmid. The first PCR product was amplified using primers CMVRNA2‐1893F (with *Hin*dIII) and the reverse primer specific for CMVΔ2b (3CC2b2419R); the second PCR product was amplified using RNA2‐2471F and CMVRNA2R (with a *Pst*I site) on the PCR template pFny209. The third PCR procedure was performed by mixing the PCR fragments of the first and second PCR procedures and involved the use of CMVRNA2‐1893F and CMVRNA2R primer pairs.

### 4.4 Yeast two‐hybrid assay

The protein–protein interaction between the 2b VSR and the cucumber RDR1 defence proteins was carried out using the GAL4‐based Matchmaker Gold Yeast Two‐Hybrid System (Clontech; Y2H). Y2H Gold and Y187 yeast strains were used for the transformation of bait and prey vectors, respectively. Small‐scale transformation was performed according to the manufacturer's protocols and plates were incubated at 28 ℃ for 3 days. Yeast mating was carried out with positive colonies of 2b and CsRDR1s as per the manufacturer's instructions. Transformants were selected on selection medium plates: SD/−Leu/−Trp/+AbA, followed by SD/−Leu/−Trp/−His/−Ade/+AbA. For the dilution assay, double‐transformants were grown in SD/−Leu/−Trp broth overnight and different dilutions at equal OD_600_ were spotted on selection plates SD/−Leu/−Trp/−His/−Ade/+AbA, followed by incubation at 28 ℃ for 3–4 days.

### 4.5 BiFC assay

In planta confirmation of interactions between 2b and CsRDR1a/1b/1c1 (with CP used as a control) was performed in *N*. *benthamiana* by BiFC assay. pSPYNE(R) 173 and pSPYCE (M) vectors were used for cloning (Waadt et al., 2008) and these BiFC constructs were transformed into the *A*. *tumefaciens* GV3101. Further agroinfiltration was carried out by resuspending *A. tumefaciens* cells in MES‐MgCl_2_ induction buffer (10 mM MES, 10 mM MgCl_2_, pH 5.6, 200 µM acetosyringone) at a final OD_600nm_ of 0.5/0.5 for target proteins and 0.3 for the p19 suppressor, using a needleless syringe. YFP fluorescence was observed in infiltrated leaves at 48 hr after treatment under a confocal microscope.

### 4.6 Protoplast isolation and transfection

Protoplasts were isolated from cucumber leaves as described previously (Owen et al., 2016), with minor modifications to the enzymolysis and transfection buffers. In the enzyme buffer, 0.5% Onozuka cellulase R10, 0.25% Onozuka macerase, bovine serum albumen (0.1%), and CaCl_2_ (10 mM) were added to MMC buffer (600 mM mannitol, 5 mM MES, 10 mM CaCl_2_, pH 5.8) and the solution was heated to 55 ℃ for 10 min. For the transfection buffer, 35% polyethylene glycol (PEG; 4,000) and 5 mM CaCl_2_ solution were used. For a single transfection, 15 µg of the respective plasmid or transcripts were used. For colocalization, 9 µg of each were used. Briefly, for transfection, 200 µl of protoplasts containing 2 × 10^6^ cells was mixed gently with plasmids (30 µl) and 500 µl of PEG (35%) was added to this mixture. The mixture was then incubated at room temperature for 5 min. Transfected protoplasts were incubated in the dark at 26 ± 2 ℃ for 68 hr.

### 4.7 Confocal microscopy

For confocal microscopy, agroinfiltrated leaf sections were cut and mounted in water under a cover glass. The protoplasts were observed on a slide containing a depression in the centre under a cover glass. Simultaneous detection of two fluorophores and chloroplast autofluorescence was performed by sequential scanning at their respective excitation and emission spectra. Excitation/emission wavelengths for GFP, CFP, YFP, and the chloroplasts were 488 nm/497–565 nm, 442 nm/450–500 nm, 514 nm/520–565 nm, and 514 nm/680–740 nm, respectively. Each fluorescent protein was scanned individually to avoid crosstalk.

### 4.8 RT‐qPCR

Total RNA was extracted from mock control and CMV‐inoculated cucumber leaf samples using TRIzol. One microgram of total RNA was treated with DNase (Thermo Fisher Scientific) according to the manufacturer's instructions. First‐strand cDNA synthesis was performed using the qPCRBIO cDNA synthesis kit (PCR Biosystems). Quantitative PCR was performed using 2.5 µl of diluted cDNA, 7.5 µl of SYBR green 2× master mix (Applied Biosystems) and 1 µl of forward and reverse primer (3 µM each), in a final volume of 15 µl of reaction mixture in a StepOne Real‐Time system (Applied Biosystems). The reaction conditions included denaturation at 94 ℃ for 20 s, followed by 40 cycles of 94 ℃ for 3 s and 60 ℃ for 30 s. The host *EF‐1α* gene was used as an internal control for cDNA normalization. For each replicate, total RNA was extracted from pooled samples of three test plants. Relative expression was quantified using the 2^−ΔΔ^ *^C^* ^t^ method. Three biological replicate leaf discs (50 mg fresh weight) were taken for analysis and each sample was a pool of three plants.

### 4.9 Protein extraction and western blotting

For detection of CMV CP in inoculated plants, total protein was extracted from plant samples using urea‐SDS extraction buffer (7.5 mM Tris‐HCl pH 6.8, 9 M urea, 4.5% sodium dodecyl sulphate [SDS], 7.5% β‐mercaptoethanol). The homogenized samples were incubated at 100 ℃ for 10 min and then centrifuged in a microfuge at maximum speed for 10 min. The supernatant was analysed on 12% polyacrylamide gel. Three biological replicate leaf discs (50 mg fresh weight) were taken for analysis and each sample was a pool of three plants.

## CONFLICT OF INTEREST

Authors declare no competing financial interest.

## Supporting information


**FIGURE S1** Interaction of *Cucumber mosaic virus* (Fny strain) movement protein (MP) with cucumber RNA‐dependent RNA polymerase 1 proteins (CsRDR1) in yeast cells. Yeast mating was performed between MP and CsRDR1 transformants, and transformants were selected on double‐dropout selection (DDO) media plates that were kept at 28 ℃ for 3 days. The dilution assay was carried out on DDO, triple‐dropout (TDO), and quadruple‐dropout (QDO) selection plates, up to 10^−4^ dilution, which were kept for 3‒4 days before they were photographed

**FIGURE S2** Screening for autoactivation of 2b (CMV‐Fny strain) constructs in the yeast two‐hybrid Gold system. Full‐length 2b and a number of 2b deletion protein baits (2b_1‐61_, 2b_38‐110_, 2b_62‐110_, 2b_1‐95_, 2b_38‐95_, and 2b_62‐95_) transformed into Y2H Gold yeast cells, which were mated with Y187 yeast cells carrying the empty vector (control; pGADT7). Diploid transformants were selected on double‐dropout (DDO) selection plates. Further dilution assays were performed on DDO, triple‐dropout (TDO/AbA), and quadruple‐dropout (QDO/AbA) selection plates supplemented with aureobasidin A (an antibiotic) and those plates were incubated at 28 ℃ for 3‒4 days. The positive control was pGADT7‐SV‐40 T‐antigen and pGBKT7‐p53 protein

**TABLE S1** List of primers used during the study

## Data Availability

The data that support the findings of this study are available from the corresponding author upon reasonable request.
