# Supplementary figures and images for: Cucumber RDR1s and cucumber mosaic virus suppressor protein 2b association directs host defence in cucumber plants
Source: Mol Plant Pathol. 2021 Aug 6;22(11):1317–31. doi: 10.1111/mpp.13112 (PMC8518566; doi:10.1111/mpp.13112)

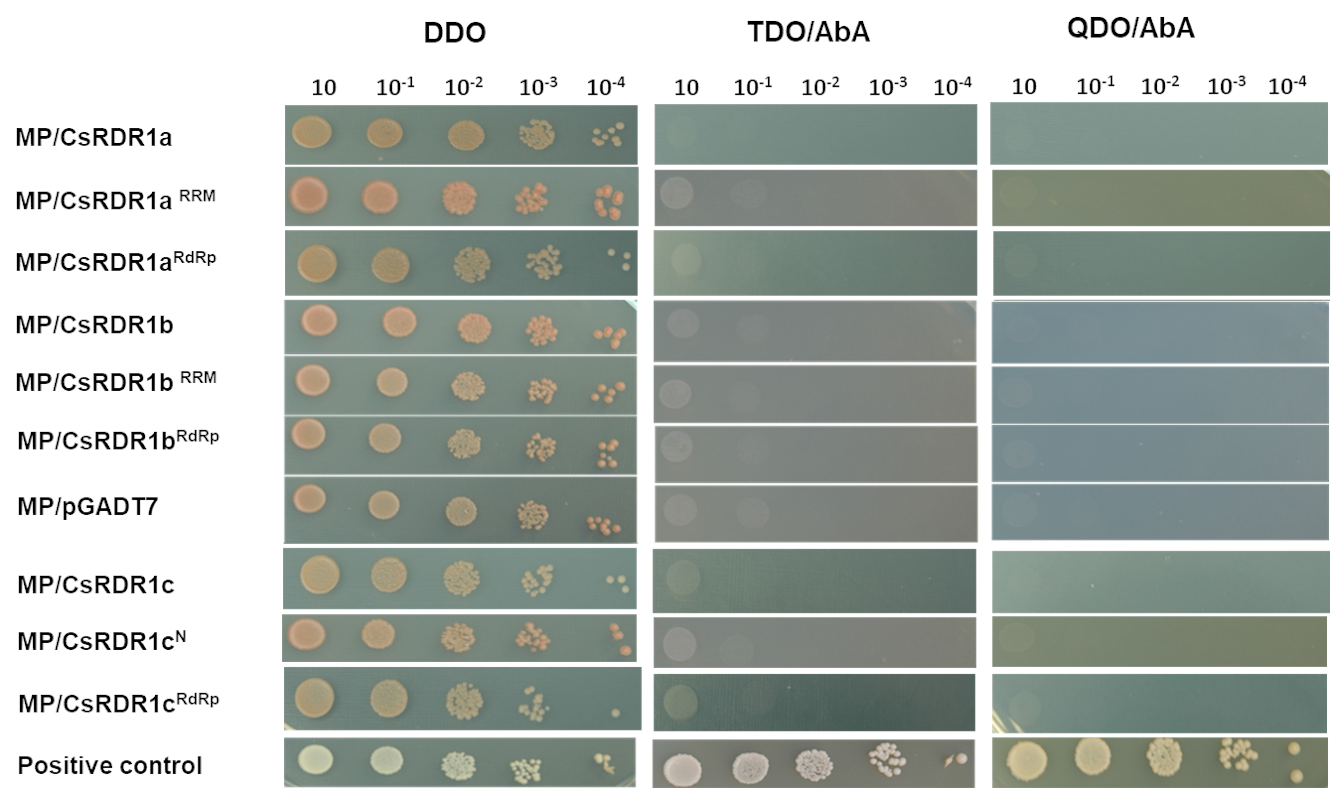

Supplement: Supplementary file 1 — FIGURE S1 Interaction of Cucumber mosaic virus (Fny strain) movement protein (MP) with cucumber RNA‐dependent RNA polymerase 1 proteins (CsRDR1) in yeast cells. Yeast mating was performed between MP and CsRDR1 transformants, and transformants were selected on double‐dropout selection (DDO) media plates that were kept at 28 ℃ for 3 days. The dilution assay was carried out on DDO, triple‐dropout (TDO), and quadruple‐dropout (QDO) selection plates, up to 10−4 dilution, which were kept for 3‒4 days before they were photographed [file MPP-22-1317-s001.tif]

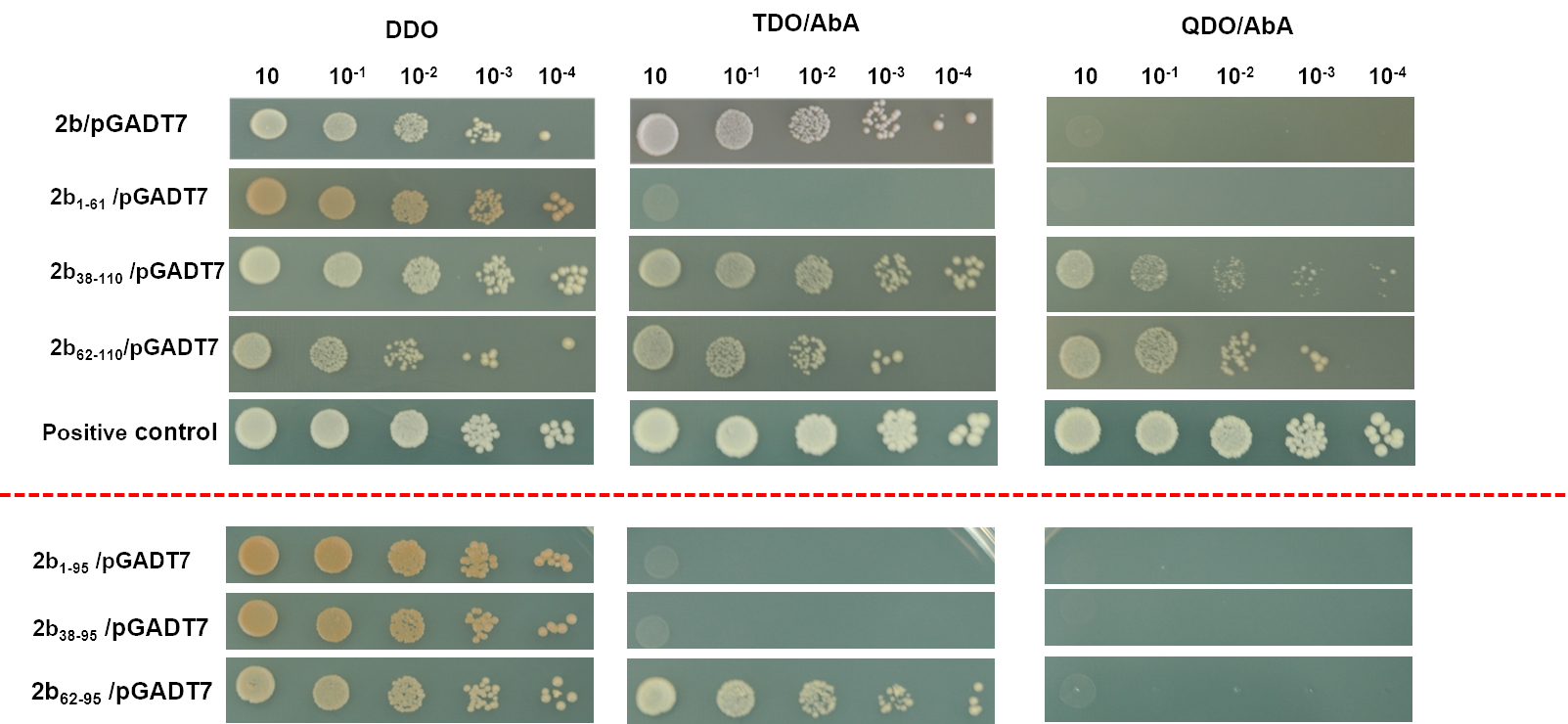

Supplement: Supplementary file 2 — FIGURE S2 Screening for autoactivation of 2b (CMV‐Fny strain) constructs in the yeast two‐hybrid Gold system. Full‐length 2b and a number of 2b deletion protein baits (2b1‐61, 2b38‐110, 2b62‐110, 2b1‐95, 2b38‐95, and 2b62‐95) transformed into Y2H Gold yeast cells, which were mated with Y187 yeast cells carrying the empty vector (control; pGADT7). Diploid transformants were selected on double‐dropout (DDO) selection plates. Further dilution assays were performed on DDO, triple‐dropout (TDO/AbA), and quadruple‐dropout (QDO/AbA) selection plates supplemented with aureobasidin A (an antibiotic) and those plates were incubated at 28 ℃ for 3‒4 days. The positive control was pGADT7‐SV‐40 T‐antigen and pGBKT7‐p53 protein [file MPP-22-1317-s003.tif]
