# Supplementary material for: Cucumber RDR1s and cucumber mosaic virus suppressor protein 2b association directs host defence in cucumber plants
Source: Mol Plant Pathol. 2021 Aug 6;22(11):1317–31. doi: 10.1111/mpp.13112 (PMC8518566; doi:10.1111/mpp.13112)
Supplement: Supplementary file 3 — TABLE S1 List of primers used during the study [file MPP-22-1317-s002.docx]

Table S1: List of primers used during the study

|  | **For Y2H constructs** |
| --- | --- |
| CMV 2b | F 5’CATATGGAATTGAACGTAGGTGCAATGAC3’  R 5’GGATCCTCAGAAAGCACCTTCCGCCCATTC3’ |
| CMV CP | F 5’CATATGGACAAATCTGAATCAACCAGTGC3’  R 5’GAATTCTCAGACTGGGAGCACTCCAGATGTG3’ |
| CMV MP | F 5’CATATGGCTTTCCAAGGTACCAGTAGGAC3’  R 5’GAATTCCTAAAGACCGTTAACCACCTGCGG3’ |
| CsRDR1b | F 5’CATATGGGTAAAACAATTCAGCTTTTTGG3’  R 5’GAATTCTCAATAGCGAGCTCTACAATTAAT3’ |
| CsRDR1b^RRM^ | F 5’CATATGATTCAGCTTTTTGGATTCCCTTC3’  R 5’GAATTCTTACTTAGATATAATTGACTTAGC3’ |
| CsRDR1b^RdRp^ | F 5’CATATGGGTCCAGAAGTTAACATTTCAAATC3’  R 5’GAATTCCTAGACTGCAATTGAGAATAGTTTTG3’ |
| CsRDR1a | F 5’CCCGGGATGGGGAAAACAATTCACATTAGTGG3’  R 5’GGATCCTCAACTCCCACCAAGATTTAACAC3’ |
| CsRDR1a^RRM^ | F 5’CATATGATTCACATTAGTGGATTTCCTTC3’  R 5’GAGCTCTCATCCGTACCATAGACGTTGATT3’ |
| CsRDR1a^RdRp^ | F 5’GCTCATATGCCTTGTAAGGTGTTCTTCTGTGG3’  R 5’CAGCTCGAGTTACACAGCCCGAAAAAGTTTTC3’ |
| CsRDR1c | F 5’CATATGGGAAAGACGATCGAAATTTACG3’  R 5’GAGCTCTCACCCCATCAAAATTGTGTGTAATA3’ |
| CsRDR1c^RRM^ | F 5’CATATGGGAAAGACGATCGAAATTTACG3’  R 5’GAGCTCTCATTTCCTCTCCTCCGTTGTCG 3’ |
| CsRDR1c^RdRp^ | F 5’ CATATGCCGTTGAAAGTGTACTTCTGTG3’  R 5’ GAGCTCCACTCCTCTAAAAAGCTTCCC3’ |
|  | **For BiFC constructs** |
| CMV 2b | F 5’ GGATCCATGGAATTGAACGTAGGTGCAATG3’  R 5’ CTCGAGGAAAGCACCTTCCGCCCATTCG3’ |
| CMV CP | F 5’GGATCCATGGACAAATCTGAATCAACCAG3’  R 5’CCCGGGGACTGGGAGCACTCCAGATGTG3’ |
| CsRDR1a | F 5’GTGGATCCATGGGGAAAACAATTCACATTAGTGG3’  R 5’ATCCCGGGACTCCCACCAAGATTTAACACATG3’ |
| CsRDR1b | F 5’ GGATCCATGGGTAAAACAATTCAGCTTTTTG3’  R 5’ CTCGAGTCAATAGCGAGCTCTACAATTAAT3’ |
| CsRDR1c | F 5’CGGGATCCATGGGAAAGACGATCGAAATTTAC3’  F 5’ AGGAGCTCTCACCCCATCAAAATTGTGTGTAA3’ |
|  | **For pSAT constructs** |
| CMV 2b | F 5’ATCTCGAGCTATGGAATTGAACGTAGGTGCAA3’  R 5’GTGGATCCGAAAGCACCTTCCGCCCATTCG3’ |
| CMV CP | F 5’CCATGGACAAATCTGAATCAACCAGTGCTG3’  R 5’GGATCCGACTGGGAGCACTCCAGATGTGGG3’ |
| CsRDR1a | F 5’GCAGTCGACATGGGGAAAACAATTCACATTAGT3’  R 5’GGTGGATCCACTCCCACCAAGATTTAACAC3’ |
| CsRDR1b | F 5’ATCTCGAGCTATGGGTAAAACAATTCAGCTT3’  R 5’GTGGATCCATAGCGAGCTCTACAATTAATTTTG3’ |
| CsRDR1c | F 5’TCGAATTCTATGGGAAAGACGATCGAAATTTAC3’  R 5’GGTGGATCCTCACCCCATCAAAATTGTGTGT3’ |
|  | **For infectious clone** |
| RNA2 1893 FP | GAAAAGCTTTTATTCTCAGGCGATG |
| RNA2 RP | CAACTGCAGTGGTCTCCTTTTGGAGGCCC |
| 3CC2b2419R | GTGGTTGCACCTACGTTCAATTCGGTAATTCTTTCGCTGTTTG |
| RNA2-2471F | ACCGAATTGAACGTAGGTGCAACCAC |
|  | **For Real Time** |
| EF-1α | F 5’GAACAAGAGGTCATTCAAGTATGCT3’  R 5’CTGTGAGGTTCCAGTAATCATGTT3’ |
| CsRDR1a | F 5’CTGCACCCTGGTGATGTTC3’  R 5’GGATGAGGCCTTGATCCTTT3’ |
| CsRDR1b | F 5’TAACAGCCGTGGATGTACCA3’  F 5’ATCGCTTCCAGAGCATTCAT3’ |
| CsRDR1c | F 5’GGAAGCTTTTTAGAGGAGTGAAAGA3’  R 5’CCCCAACTTGAAATCGTACTTAGTT3’ |
| CMV CP | F 5’CTATTAACCACCCAACCTTTGTAGG3’  R 5’AGCTTCTTATCATATTCCGTGACTG3’ |

Restriction sites are underlined.
